# Management and short-term outcomes of neonates born to mothers with active perinatal SARS-CoV-2 infection

**DOI:** 10.1186/s12887-021-02872-0

**Published:** 2021-09-13

**Authors:** Vineet Lamba, Joan Lien, Jay Desai, Ajay J. Talati

**Affiliations:** 1 Division of Neonatology, Department of Pediatrics, University of Tennessee Health Sciences Center, Memphis, TN USA; 2 Department of Pediatrics, University of Tennessee Health Sciences Center, Memphis, TN USA

## Abstract

**Objective:**

We report here on the management and outcomes of neonates born to mothers with active perinatal SARS-CoV-2 infection.

**Study design:**

In this prospective study, eligible neonates were enrolled in a database to track in-hospital outcomes and followed up outpatient periodically till 2 months of age to assess for late onset symptoms of infection.

**Results:**

From April 2020 to February 2021, 67 mothers with perinatal SARS-CoV-2 infection and 70 at-risk neonates were included. Two neonates (3%) tested positive for SARS-CoV-2 within 48 h of life but remained asymptomatic during hospitalization and at all follow-up periods. Three infants were reported to have a febrile illness in 2 months follow up period, none of which was attributable to SARS-CoV-2.

**Conclusion:**

Our data supports the emerging evidence which describes a probable low risk of vertical transmission of SARS-CoV-2. We also demonstrate a low risk of post-natal transmission or late-onset symptomatic infection with SARS-CoV-2.

## Introduction

The coronavirus disease (COVID-19) caused by novel severe acute respiratory syndrome coronavirus 2 (SARS-CoV-2) has been globally spreading since the end of December 2019. Historically when looking at previous coronavirus pandemics caused by SARS-associated coronavirus (SARS-CoV) and Middle Eastern respiratory syndrome coronavirus (MERS-CoV), there were reported cases of spontaneous abortions, preterm births, low birth weight, and neonatal respiratory infections [1]. This was hypothesized to be a result of increased inflammatory activity associated with the first and third trimesters and a period of overall decreased immune activity associated with the second trimester and in the newborn period [2, 3]. At the beginning of the COVID-19 pandemic, this prior experience from pandemics caused by the same family of viruses, raised the concern that pregnant women and newborns could be at high risk for SARS-CoV-2 related complications.

Early reports of pregnant women infected with SARS-CoV-2 in the third trimester raised concern for increased risk of premature delivery [4], while large cohorts of pregnant women in the UK suggested that they are not at increased risk of spontaneous abortion or preterm birth but do have higher rates of Cesarean section [5–7]. In a review of 262 pregnant women who were admitted with confirmed COVID-19 and delivered, most were admitted due to symptoms in their 3rd trimester, 75% gave birth at that time, 59% had a Cesarean section (C-section), and 5% of neonates tested positive for SARS-CoV-2 but did not have evidence of severe illness [6]. In a review of literature published on COVID-19 in pregnant women worldwide (primarily China and US), it was reported that most women presented in their second and third trimesters, with 65–88% developing mild to moderate pneumonia and 64–78% undergoing C-sections [8, 9]. A more recent report from the INTERCOVID multinational cohort has shown and increased overall maternal morbidity in pregnant women with COVID-19 and increased risk for preterm birth, severe neonatal and perinatal morbidity indices [10]. In addition to this overall increase in maternal morbidity, the placenta may be affected as well [11] which raised the concern for possibility of vertical transmissions [12]. More recently some variants, particularly the SARS-CoV-2 B.1.617.2 variant (delta variant) have been reported to have been associated with more severe infection and worse pregnancy outcomes [13].

Initial review of SARS-CoV-2 positive neonates suggested that newborns were at risk for SARS-CoV-2 related complications [8]. De Bernardo et al. reviewed 18 studies from December 2019 to April 2020 and found that 21 of 25 neonates positive for COVID-19 were symptomatic, with 32% requiring ICU admission and 20% requiring mechanical ventilation. The most common symptoms were fever, vomiting, and cough or shortness of breath. Major complications reported were pneumonia or respiratory distress, and there were no reported deaths [14].

With increasing concern for COVID-19 related complications, the question of whether there was any risk of vertical transmission from SARS-CoV-2 infected mother to fetus and newborn came to light. It is well-established that the main routes of transmission are via respiratory droplets and contact [15, 16]. Neonates stand additional risk of infection by possible intrauterine transmission, intrapartum infection after exposure to maternal infected secretions or feces around the time of birth and postpartum infection from infected mother. Although the majority of reports have not shown any strong evidence of vertical transmission of SARS-CoV-2 [17–19], there have been some anecdotal cases with possible vertical transmission based on neonatal nasopharyngeal RT-PCR swab or presence of IgG and IgM antibodies for SARS-CoV-2 [20–22].

While there is growing literature describing low risk of vertical transmission of this virus to newborns, there is still concern for intrapartum transmission through infected maternal infected secretions or feces at time of birth or post-natal transmission from respiratory secretions of infected mother [23]. Timely initiation of appropriate precautions to decrease risk of transmission became imperative. Recommendations for the most appropriate management regarding isolation, breastfeeding, and discharge practices have evolved through the pandemic. It is not well-established what the best practices will minimize risk of post-natal infection [23]. Additionally, given the incubation period, there is the consideration of whether neonates will develop late-onset infections or sequelae after hospital discharge in those born to mothers with active infection. We sought to review the outcomes of infants born to mothers with active perinatal COVID-19 infection during initial hospital stay and up to 2 months of age.

## Methods

### Study design and participants

We established a database to prospectively enroll all neonates born to mothers with active perinatal COVID-19 infections presenting to our network of two neonatal intensive care units (NICUs) starting in April 2020. The network includes a referral level 4 center in a freestanding children’s hospital and a level 3 unit located in a regional perinatal center in Memphis, TN. Active perinatal SARS-CoV-2 infection was defined as maternal infection diagnosed between 14 days prior to and up to 72 h after delivery. In keeping with national practice, active infection was defined by detection of SARS-CoV-2 by reverse transcription polymerase chain reaction (RT-PCR) on nasopharyngeal (NP) swabs. Initially, testing for mothers was symptoms-based between April and May 2020; thereafter, all mothers were uniformly screened with NP swabs on admission for delivery with the option to opt-out unless symptomatic. If the mother was confirmed positive for SARS-CoV-2 or was a person under investigation (PUI) at time of delivery, the neonate would undergo testing at 24 and 48 h of life. Only infants of confirmed SARS-CoV-2 positive mothers were included in the database.

All data were collected by chart review and stored in a secure HIPPA compliant database. Institutional review board (IRB) approval was obtained from the UTHSC IRB and the hospital research boards.

### Statistical analysis

Chi squared test of independence was used to compare outcomes of prematurity and c-section were compared between woman with and without active perinatal SARS-CoV-2 infection. All analyses were conducted using R, version 4.0.5 (R Foundation). Two-sided *P* < .05 indicated significance.

### Data collection & follow-up

**Fig. 1:**
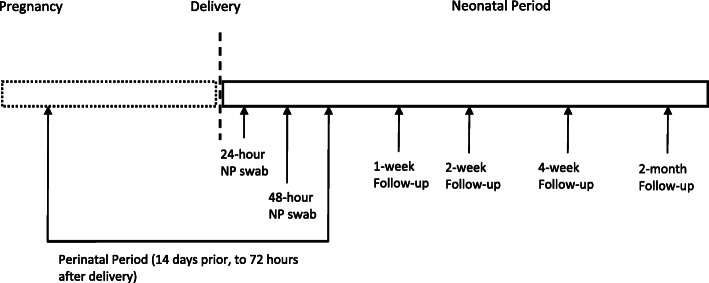
Timeline of Neonatal Testing and Follow-Up

We collected several maternal and neonatal variables, which included demographics, maternal symptoms, treatment, mode of delivery, neonatal symptoms, treatment, mode of feeding, and lab data when available. We also collected limited data on the control cohort i.e. deliveries during study time period with no evidence of active maternal perinatal infection. Follow-up of infants was performed by telephone calls at 1-, 2-, 4-weeks and 2 months of age if discharged to assess for any late onset symptoms (Fig. 1).

### Delivery room practices

In order to minimize exposure and conserve personal protective equipment (PPE), we re-classified delivery room attendance criteria to allow the pediatric members of delivery team to remain on “stand-by.” When attendance was required, if feasible, a single team member would perform an initial assessment of the neonate before remaining team members would enter if indicated. For all deliveries of PUIs or confirmed SARS-CoV-2 positive mothers, PPE based on Centers for Disease Control and Prevention (CDC) and American Academy of Pediatrics (AAP) recommendations was used (i.e., N95 mask, eye shield, gown, and gloves). Given lack of data regarding transplacental transmission, routine delayed cord clamping was continued; however, skin-to-skin contact immediately after birth was avoided. Mothers and any family members present were required to wear surgical face masks during deliveries.

### Isolation practices & neonatal care

As national data and recommendations evolved, so did our separation practices. From April to September 2020, any neonate born to a PUI or confirmed SARS-CoV-2 positive mother was routinely separated and isolated in the NICU until negative maternal testing in the case of PUIs or until discharge in the case of confirmed positive mothers. After September 2020, mothers were considered eligible and given the option for rooming-in if they were asymptomatic, with no need for respiratory support, afebrile with stable vital signs, and able to care for their baby. Neonates were eligible for rooming-in if they were well-appearing, with no need for respiratory support, able to feed without additional support, and had normal vital signs. Practice for symptomatic SARS-CoV-2 positive mothers remained unchanged.

When separated in the NICU, neonates were placed in closed incubators (isolettes) and cohorted in a single large isolation room with isolettes placed 6 ft apart. When rooming-in with mother, the neonate was kept in an isolette at least 6 ft away from mother’s bed, and mothers were instructed to wear masks and to perform hand hygiene prior to and after taking care of the infant (including breastfeeding). Breastmilk feeding (directly or expressed) was always encouraged after counseling mother of unknown but likely exceptionally low risk of transmission by breastmilk. In both settings, medical providers interacting the neonates used Airborne, Droplet, and Contact Precautions-level PPE. Neonates were bathed soon after birth and underwent testing with NP RT-PCR swabs at 24 and 48 h of life while those in the newborn nursery or rooming-in generally only received testing at 24 h of life prior to discharge. Newborns in the NICU were removed from isolation after two negative tests.

Given the possible increased risk of aerosol generation, if neonatal SARS-CoV-2 status was unknown or positive and they required non-invasive respiratory support greater than 2 l per minute of high flow nasal cannula, they were intubated with HEPA filter placed in expiratory limb of ventilator circuit. These infants were placed in negative pressure room when available.

Regarding discharge practices, when possible, infants were discharged to a family member with a proven negative SARS-CoV-2 test and families were educated to isolate from mother for 10 days from her positive test. If no family member was available and mother was symptomatic, infant was kept in NICU for 7 days then discharged home with mother with education on handwashing/breast hygiene and continuation of droplet precautions.

## Results

Between April 2020 to April 2021, there were 2367 deliveries, of which 67 mothers tested positive for SARS-CoV-2 via NP RT-PCR in the perinatal period. Of these women, 70 neonates were identified, with 65 single, 1 twin, and 1 triplet gestation live-born deliveries.

Demographic and clinical characteristics of the mothers are provided in Table 1. Forty-four (66%) mothers identified as African American, 6 (9%) as Caucasian, and 15 (22%) Hispanic. Mean maternal age was 26.7 ± 6.4 years old. Of the 67 mothers, 54 (81%) were asymptomatic before admission, while 9 (13%) reported being sick at home and 4 (6%) required hospitalization for SARS-CoV-2 within 14 days prior to delivery. Forty-two (63%) mothers were confirmed COVID-19 positive at the time of delivery, the remainder were persons under investigation (PUIs) at time of delivery and later confirmed to be positive. Overall, median time from diagnosis to delivery was 1 day (IQR, 0 to 1.75 days), with a positive number indicating that the test was obtained before birth. Maternal indications for testing are reported in Table 1–55 (89%) of women underwent asymptomatic screening and 4 (6%) had previous positive tests. The remaining were tested due to symptoms that included fever, upper or lower respiratory symptoms, anosmia or ageusia, or recent known SARS-CoV-2 contact.

**Table 1:** Characteristics of Mothers with SARS-CoV-2 Infection

Characteristic	No. (%)
**Number of mothers**	67
**Age, median (IQR), in years**	27 (21–31)
**Ethnicity**	
White	6 (9%)
Hispanic	15 (22%)
Black or African American	44 (66%)
Other	2 (3%)
**Mode of delivery**	
Vaginal	39 (58%)
Cesarean	28 (42%)
**Indications for Cesarean delivery**	
Repeat C-section.	10 (36%)
Worsening maternal condition due to COVID-19	3 (11%)
Pre-eclampsia	3 (11%)
Chorioamnionitis	2 (7%)
Arrest of descent	2 (7%)
Malpresentation	3 (11%)
Fetal distress	10 (36%)
Severe IUGR	2 (7%)
Other	5 (18%)
**Maternal condition before admission**	
Asymptomatic	54 (81%)
Sick at home	9 (13%)
Required hospitalization within 14 d prior to delivery	4 (6%)
**Indication for SARS-CoV-2 testing**	
URI symptoms	3 (5%)
LRI symptoms	8 (13%)
Fever	3 (5%)
Gastrointestinal symptoms	0 (0%)
Myalgias or fatigue	0 (0%)
Anosmia or ageusia	2 (2%)
Contact with SARS-CoV-2 case	3 (5%)
Previous positive	4 (6%)
Asymptomatic screening	55 (89%)
**SARS-CoV-2 status at time of delivery**	
Confirmed positive.	42 (63%)
Person under investigation (PUI)	25 (37%)
**Time from diagnosis to delivery, median (IQR), in days**	1 (0–1.75)
**Specific treatment for SARS-CoV-2**	
None	61 (91%)
Remdesivir	3 (5%)
Dexamethasone	4 (4%)
Convalescent plasma	1 (2%)
**Length of stay, median (IQR), in days**	2 (2–3)
**Final maternal disposition**	
Discharged home	67 (100%)
Death	0 (0%)

*Abbreviations*: *IQR* interquartile range, *SARS-CoV-2* severe acute respiratory syndrome coronavirus 2, *IUGR* In utero growth restriction, *URI* Upper respiratory tract infections, *LRI* Lower respiratory tract infections

Of the 13 symptomatic mothers, 6 received specific treatment for COVID-19. Three received Remdesivir, two of them also received dexamethasone, and of those, one received convalescent plasma therapy as well due to severe disease. Two mothers received a combination of dexamethasone, zinc, and vitamin D; and one mother was treated with zinc and vitamin D. There were no maternal deaths related or non-related to COVID-19.

In the cohort, 15 (21%) had preterm births and 42% underwent C-section, however we did not see any statistically significant difference in preterm birth (15 of 67 [21%] vs 534 of 2300 [23.2%]; *p*-value = 0.87) or C-section (28 of 67 [42%] vs 805 of 2300 [35%]; *p*-value = 0.25) between mothers with and without perinatal SARS-CoV-2 infection during the study period.

Of the 70 neonates enrolled in the study, 37 (53%) were female, 55 (79%) were born at term (≥37 weeks gestational age), and mean gestational age was 37.3 ± 2.9 weeks (median 38 weeks, range 28–41 weeks). There were 28 (42%) cesarian deliveries. The most common maternal indications for C-sections were repeat C-section (*n* = 10) and 3 underwent C-section for worsening maternal condition due to COVID-19. Fetal indications for C-section included fetal distress (*n* = 10), malpresentation (*n* = 3), and severe intrauterine growth restriction (*n* = 2). Mean birth weight was 2988 ± 733 g. Median APGAR score at 5 min was 9 (range, 4–10). Median (IQR) lengths of maternal and neonatal hospitalization were 2 (2–3) days and 3 (2–6) days, respectively. Characteristics of the neonates are summarized in Table 2.

**Table 2:** Characteristics of Neonates Born to SARS-CoV-2-Infected Mothers

Characteristic	No. (%)
**Number of neonates**	70
**Sex**	
Male	33 (47%)
Female	37 (53%)
**Gestational age, mean (SD), in weeks**	37.3 (2.9)
**Birth weight, mean (SD), in grams**	2988 (733)
**APGAR score at 5 min, median (range), in points**	9 (4–10)
**Prematurity**	
Preterm (less than 37 completed weeks)	15 (21%)
Term (greater than 37 weeks)	55 (79%)
**Resuscitation at birth**	
Drying and stimulation	70 (100%)
Oxygen supplementation	21 (32%)
Positive pressure ventilation	20 (31%)
Intubation	6 (9%)
**Maximum respiratory support during hospitalization**	
None	53 (76%)
Supplemental oxygen	2 (3%)
Continuous positive airway pressure	4 (6%)
Mechanical ventilation	10 (14%)
Extracorporeal membrane oxygenation	1 (2%)
**Type of feeding**	
Directly breast fed by mother	7 (11%)
Expressed breast milk fed by another caregiver	5 (8%)
Formula-fed	58 (92%)
**Length of stay, median (IQR), in days**	3 (2–6)
**Received SARS-CoV-2 RT-PCR testing during first 14 d of life**	69 (99%)
At birth	2 (3%)
At DOL 1	65 (93%)
At DOL 2	49 (70%)
At DOL 3	5 (7%)
Between DOL 4 and 14	2 (2%)
**SARS-CoV-2 positivity**	
At birth	0 (0%)
At DOL 1	1 (2%)
At DOL 2	1 (2%)
At DOL 3	0 (0%)
Between DOL 4 and 14	0 (0%)
**Final neonatal disposition**	
Discharged home	68 (97%)
Transferred to another facility	2 (3%)
Death	0 (0%)

*Abbreviations*: *SD* standard deviation, *IQR* interquartile range, *SARS-CoV-2* severe acute respiratory syndrome coronavirus 2, *DOL* day of life

In addition to the standard drying and stimulation during resuscitation at delivery, 21 (32%) neonates needed supplemental oxygen delivered, 20 (31%) received positive pressure ventilation, and 6 (9%) were intubated. Maximum respiratory support during neonatal hospitalization was tracked, and while the majority did not require any respiratory support (*n* = 53, 76%), two (3%) required supplemental oxygen, four (6%) were on continuous positive airway pressure, 10 (14%) were mechanically ventilated, and one (1%) was placed on extracorporeal membrane oxygenation (ECMO). The neonate that was placed on ECMO required it for management of meconium aspiration syndrome. We reviewed every case requiring mechanical ventilation and respiratory support, all cases had independent risk factors predicting need for respiratory support, most commonly prematurity (7 of 10 neonates mechanically ventilated were preterm). All neonates requiring supplemental oxygen or additional respiratory support were negative for SARS-CoV-2.

Of the 70 mother and infant pairs, 46 (66%) were separated at birth due to positive maternal SARS-CoV-2 status (given our initial protocol requiring separation for all positive mothers), complications with delivery or pending maternal convalescence, or if neonate required increased resuscitation efforts or support. A total of 30 (46%) neonates roomed-in with mothers during their hospitalization, and of these, 18 (60%) were also admitted to the NICU during their hospital stay. Of the 18 newborns, 5 (29%) were initially roomed-in but were transferred to the NICU due to development of maternal complications.

A total of 123 NP swabs were obtained. Sixty-nine of 70 (99%) neonates were tested during the first 14 days of life. Two (3%) were tested on day of life 0 (day of birth), 65 (93%) were tested on day of life (DOL) 1, 49 (70%) on DOL 2, 5 (7%) on DOL 3, 1 (2%) on DOL 4, and 1 (2%) on DOL 9. Of these, 67 (97%) neonates tested negative. Of the two who tested positive, one (neonate A) was positive on DOL 1 with repeat testing on DOL 2 being negative, and the other (neonate B) was positive on DOL 2 after a negative test on DOL 1. Neonate A was born to an asymptomatic 16-year-old G1 at 36 weeks gestational age via spontaneous vaginal delivery, length of stay was 2 days, and was found to be asymptomatic at till 2 months of age. Neonate B was born to an asymptomatic 20-year-old G2 at 37 weeks gestational age via spontaneous vaginal delivery, length of stay was 3 days, and was found to be asymptomatic till 2 months of age as well.

Follow-up response rates were 90, 90, 89, and 83% at 1-, 2-, 4-week, and 2-month time periods, respectively. Of the respondents, all neonates were asymptomatic at the 1-week follow-up. At the 2-week follow-up, one neonate was reported to have be febrile secondary to a urinary tract infection with no other COVID-19 related symptoms. At 4-week follow-up, one infant was admitted for 2 days after presenting with increased work of breathing and vomiting but remained afebrile and SARS-CoV-2 test was negative at that time. At 2-month follow-up, there was one neonate that family reported had a self-limiting febrile illness for 3 to 4 days, no COVID-19 test was obtained. A summary of follow-up data is found in Table 3.

**Table 3:** Follow-up Data

Characteristic	No. (%)
**At 1-week of age**	
Response rate^a^	63 (90%)
Symptomatic	0 (0%)
Asymptomatic	63 (100%)
**At 2-weeks of age**	
Response rate^a^	63 (90%)
Symptomatic	1 (2%)
Asymptomatic	62 (98%)
**At 4-weeks of age**	
Response rate^a^	62 (89%)
Symptomatic	1 (2%)
Asymptomatic	61 (98%)
**At 2-months of age**	
Response rate^a^	58 (83%)
Symptomatic	1 (2%)
Asymptomatic	57 (98%)

^a^Response rate percentage reported out of 70 newborns. Remainder of percentages reported based on total number of respondents

## Discussion

While case reports of SARS-CoV-2 positive newborns demonstrated significant symptoms or complications, rate of transmission has been reported to be low (0–5%), especially with appropriate precautions in place. Our 2.8% rate of positive SARS-CoV-2 infection in neonates is similar to earlier reports in literature [6]. Breslin et al. performed a retrospective review of a 15-day period describing neonatal outcomes of 18 infants who were born to mothers with confirmed COVID-19, where all neonates tested negative though timing of the testing was not reported [24]. Dumitriu et al. reported negative results of 101 neonates, with testing ranging from DOL 0 to 25 based on symptoms [18]. De Sousa et al. performed a systematic review on risks of infections among pregnant women and subsequent fetal transmission, where 75–99% of mothers were reported to have mild to moderate pneumonia, rates of C-section ranged from 64 to 78%, and there were 2 newborns who tested positive for SARS-CoV-2 out of a total of 145 newborns tested [9].

Of the two neonates who tested positive in our study, both had one negative and one positive test result in the first 48 h. It should be noted that the RT-PCR testing for SARS-CoV-2 is highly specific but not as sensitive, so the neonates were treated as positive regardless of the negative result. Both mothers of the positive neonates were asymptomatic at presentation. Neither neonates required additional resuscitation efforts other than drying and stimulation and did not have any oxygen requirements during hospital stay (length of stays were 2 and 3 days). De Bernardo et al. reviewed studies describing clinical presentations of 25 SARS-CoV-2 positive neonates, the majority of which presented with symptoms, but were found to have a good outcome with low rate of severe complications and no deaths reported. Our study also supports these findings. Additionally, in our study, the neonates who tested positive were found to be asymptomatic at all follow-up periods up to 2 months.

Of the 70 neonates, 30 (46%) roomed-in with their mothers, none of whom tested positive. During the time when all newborns were admitted to the NICU for isolation, patients were primarily formula-fed due to isolation efforts even when mothers were encouraged to pump breastmilk. However, as guidelines evolved, and direct breastfeeding was encouraged with enforcement of good hand hygiene and wearing a mask during breastfeeding. Overall, there were relatively small numbers of patients who received breast milk (17%) or were directly breastfed (11%). Of the patients who received breast milk, none tested positive for SARS-CoV-2 during hospitalization and remained asymptomatic during follow-up. There have been sporadic reports of detection of SARS-CoV-2 RNA in breastmilk, however, the presence of viral particles does not imply the possibility of transmitting infection [23].

Recent multinational cohort of 706 pregnant women with COVID-19 diagnosis showed that there was significantly higher risk for preeclampsia/eclampsia, C-sections, maternal mortality, preterm birth, severe neonatal morbidity index and neonatal complications when compared to women without COVID-19 [10]. In our cohort, we did not see a statistically significant difference in the rates of preterm birth and C-sections. There were no neonatal or maternal mortalities and when individual cases of neonates requiring mechanical ventilation were analyzed, there was an identifiable etiology for need for mechanical ventilation in all cases.

Lavizzari et al. compared international guidelines for managing neonates and found that most countries suggested viral testing on all neonates born to SARS-CoV-2 positive mothers regardless of symptoms. All countries reported limited family access to the NICU and prohibited entrance of symptomatic visitors [25]. Ronchi et al. evaluated safety of rooming-in and risk of postnatal transmission and concluded that risk of mother-to-infant transmission of SARS-CoV-2 during rooming-in was unlikely if the infected mothers were not severely affected and were educated to observe droplet and contact precautions when caring for or breastfeeding infants [26]. We believe that our guidelines which were consistent with international recommendations promoted a low rate of viral transmission.

Most studies present results of infection rate and SARS-CoV-2 positivity of neonates, however very few have described that protocols followed for the data reviewed [26]. Additionally, the furthest follow-up reported was 14 to 20 days post-discharge [26, 27]. We believe ours is the first study that reports infectivity and follow-up as far as 2 months after discharge. In our cohort, there were 3 infants reported to have a febrile illness up to 2 months of age, none directly attributable to a SARS-CoV-2 infection. Overall, our results showed an extremely low percentage of symptomatic infants at each follow-up period, which demonstrates a low-risk of late-onset infection or late presentation of vertically acquired infection after hospital discharge.

Limitations of this study included the fact that while the neonates were followed up to 2 months after discharge, they did not undergo repeat testing. With the latency of SARS-CoV-2 infection and prolonged incubation period, it is possible that the RT-PCR could still result in a positive test within 14 days of birth if there was vertical transmission. However, it would be difficult to differentiate if the transmission were vertical or horizontal if the neonates tested positive after the immediate post-natal period. Other limitations include the relatively small number of study participants and our laboratories did not perform typing of the SARS-CoV-2 virus, hence, we were unable to collect data regarding the dominant variant.

## Conclusion

Our data supports the previous findings that describe a probable very low-risk of vertical transmission of SARS-CoV-2. Additionally, with follow-up data suggests there is likely low-risk of post-natal transmission of SARS-CoV-2 or late-onset symptomatic infection or its sequelae. While there are still many unknowns with respect to controlling this pandemic, we believe that the hospital guidelines we described regarding rooming-in, isolation, breastfeeding, and discharge practices are effective for minimizing risk of transmission while still promoting family centered care.

## Data Availability

The datasets used and/or analyzed during the current study available from the corresponding author on reasonable request.
